# Characterization of XR_311113.2 as a MicroRNA Sponge for Pre-ovulatory Ovarian Follicles of Goats *via* Long Noncoding RNA Profile and Bioinformatics Analysis

**DOI:** 10.3389/fgene.2021.760416

**Published:** 2022-01-03

**Authors:** Hu Tao, Juan Yang, Pengpeng Zhang, Nian Zhang, Xiaojun Suo, Xiaofeng Li, Yang Liu, Mingxin Chen

**Affiliations:** ^1^ Hubei Key Laboratory of Animal Embryo Engineering and Molecular Breeding, Institute of Animal Husbandry and Veterinary, Hubei Academy of Agricultural Sciences, Wuhan, China; ^2^ Department of Biotechnology, College of Life Sciences, Xinyang Normal University, Xinyang, China

**Keywords:** goat, ovarian follicle, lncRNA, transcriptome, miRNA sponge

## Abstract

Long noncoding RNAs (lncRNAs) were identified recently as a large class of noncoding RNAs (ncRNAs) with a length ≥200 base pairs (bp). The function and mechanism of lncRNAs have been reported in a growing number of species and tissues. In contrast, the regulatory mechanism of lncRNAs in the goat reproductive system has rarely been reported. In the present study, we sequenced and analyzed the lncRNAs using bioinformatics to identify their expression profiles. As a result, 895 lncRNAs were predicted in the pre-ovulatory ovarian follicles of goats. Eighty-eight lncRNAs were differentially expressed in the Macheng black goat when compared with Boer goat. In addition, the lncRNA XR_311113.2 acted as a sponge of chi-miR-424-5p, as assessed via a luciferase activity assay. Taken together, our findings demonstrate that lncRNAs have potential effects in the ovarian follicles of goats and may represent a promising new research field to understand follicular development.

## Introduction

Ovulation rate is an important goat reproductive trait that determines the upper limit of the female goat litter size; in turn, the growth and development of follicles in the ovary determine the ovulation rate (Cui et al., 2009). Follicle development is a very complex biological process and its regulatory mechanism warrants further study. During mammalian ovarian folliculogenesis, which involves multiple transcription factors, primordial follicles develop into pre-ovulatory follicles, followed by ovulation, which releases mature oocytes (Choi and Rajkovic, 2006; Pan et al., 2012). The Macheng black goat is an excellent goat breed that is unique to China and is characterized by pure black hair color and its breed function is higher than Boer goat (Tao et al., 2018). In the breeding production process, PMSG-hCG is usually used to treat the ewe, and then artificial fertilization. We found that after hormone treatment, the litter size of Macheng black goat remains above Boer goat. To investigate the phenotypic differences, we analyze the pre-ovulatory follicles of ewes after hormone treatment via High-throughput sequencing and analyze the development of follicles in a highly fertile local goat.

Noncoding RNAs (ncRNAs) have always been regarded as “DNA junk” without the capability to encode proteins. More recently ncRNAs, which include mainly microRNAs (miRNAs), piwi-interacting RNAs (piRNAs), and circular RNAs (circRNAs), were demonstrated to have important biological functions. They are basically derived from the transcription activity of the genome (Jin et al., 2018). Long noncoding RNAs (lncRNAs), which were recently identified as a large class of ncRNAs, are defined as ncRNAs of more than 200 bp in length with no protein-coding capacity (Mercer et al., 2009; Hung and Chang, 2010). A recent study showed that lncRNAs play multifunctional roles in a wide variety of important biological processes, including cell proliferation (Wang et al., 2017), cell apoptosis (Lu et al., 2016), genomic splicing (Parikshak et al., 2016), chromatin remodeling (Han and Chang, 2015), and transcription (McHugh et al., 2015). In addition, lncRNAs regulate the expression of miRNA by acting as molecular sponges and reducing the inhibitory effect of miRNAs on their target genes (Paraskevopoulou and Hatzigeorgiou, 2016).

During ovarian folliculogenesis, functional lncRNAs, such as growth arrest specific 5 (GAS5) (Wang et al., 2018), lncRNA HCG26 (Liu et al., 2017), lncRNA Neat1 (Nakagawa et al., 2014; Li et al., 2017), and lncRNA MALAT1 (Li et al., 2018a), have been found to regulate follicle development and regeneration through diverse mechanisms. For instance, GAS5, which is expressed in female germ-line stem cells (FGSCs) and oocytes, promotes FGSC reproduction and survival *in vitro* and is highly expressed in neonatal mouse ovaries (Wang et al., 2018). High levels of lncRNA SRA stimulate the growth of mouse follicle granulosa cells, increase the levels of estrogen and progesterone, and upregulate the expression of key enzymes (i.e., cytochrome P450 family 19, subfamily A, member 1 (CYP19A1) and cytochrome P450 family 11, subfamily A, member 1 (CYP11A1)) (Li et al., 2018b). Although miRNAs and piRNAs have been extensively studied in ovarian folliculogenesis, the role of other valuable noncoding transcripts remains to be studied. Moreover, researchers are paying increasing attention to the role of lncRNAs in follicular development, and have used high-throughput sequencing to analyze the expression pattern of lncRNAs during follicular development. Finally, the identification of the key lncRNAs for follicular development is expected.

In the present study, differentially expressed lncRNAs were detected through deep RNA sequencing (RNA-seq) in samples of pre-ovulatory follicles from Macheng black goats and Boer goats. Eight hundred and ninety-five new lncRNAs were identified in the ovarian follicles of goats. Eighty-eight lncRNAs were differentially expressed between Macheng black and Boer goats. The lncRNA expression patterns were validated using quantitative real-time polymerase chain reaction (qRT-PCR). The greatly differentially expressed lncRNA, XR_311113.2 was demonstrated to sponge chi-miR-424-5p *via* a luciferase activity assay. Therefore, in this study we examined the expression of lncRNAs by RNA-seq during goat follicle development and explored their expression profiles and functions in goat follicles.

## Materials and Methods

### Ethics Statement

All studies involving animals were conducted according to the regulation (No. 5 proclaim of the Standing Committee of Hubei People’s Congress) approved by the Standing Committee of Hubei People’s Congress, P. R. China. Sample collection was approved by the ethics committee of Hubei Academy of Agricultural Sciences. Animals were humanely sacrificed as necessary to ameliorate suffering.

### Animals and Tissues

Aged adult ewes weighing 40 kg were obtained from the goat stud farm of the Institute of Animal Husbandry and Veterinary Medicine, Hubei Academy of Agricultural Sciences, feeding with the same pattern and environment. Macheng black goats (Kidding rate and fecundity rate: 219 and 346%) have higher reproductive performance than Boer goats (Kidding and fecundity rates: 189 and 210%). Three Macheng black goats and three Boer goats exhibiting normal estrous cycles were treated with 1000 IU PMSG (SanSheng, Zhejiang, China) and 500 IU hCG (SanSheng, Zhejiang, China) as previously described (Saharrea et al., 1998; Torner et al., 1998). PMSG-hCG was used to initiate and synchronize the follicular phase. After 36 h, the PMSG-hCG stimulated ewes were slaughtered, and the ovaries of each goat in the pre-ovulatory phase were immediately removed, stored in liquid nitrogen until RNA extraction. The total RNAs were extracted from fourteen tissues (i.e., liver, spleen, heart, kidneys, small intestine, fat, ovarian folliclelungs, lungs, uterus, abomassum, muscle, reticulum, rumen and omasum) of the Boer goats.

### RNA Extraction and Qualification

A total of 5 μg of RNA was isolated from each individual sample using the TRIzol reagent (Invitrogen, MD, United States) according to the manufacturer’s protocol. The purity and quantity of the total RNA were measured using a NanoDrop instrument and agarose gel electrophoresis. RNA integrity was assessed using the RNA Nano 6000 Assay Kit of the Bioanalyzer 2100 system (Agilent Technologies, CA, United States), and only the samples showing RNA integrity number (RIN) scores higher than 6 were used in this study.

### Library Preparation for lncRNA and mRNA Sequencing

Three micrograms (μg) of RNA was used as input material. Ribosomal RNA was completely removed using the Epicentre Ribo-zero™ rRNA Removal Kit (Epicentre, WI, United States), and then sequencing libraries were generated with the NEBNext^®^ Ultra™ Directional RNA Library Prep Kit for Illumina^®^ (NEB, Ispawich, United States) following manufacturer’s recommendations. First-strand cDNA was synthesized using M-MuLV Reverse transcriptase and random hexamer primers, and second-strand cDNA was synthesized using RNase H and DNA polymerase I. After adenylation of the 3′ ends of DNA fragments, NEB Next Adaptors were ligated to prepare for hybridization. The library fragments were purified for selecting cDNA fragments (150–200 bp in length). Finally, the products were purified with an AMPure XP system (Beckman Coulter, Beverly, United States). The libraries were sequenced on an Illumina Hiseq 4000 platform, and 150 bp paired-end reads were generated. Sequencing data were deposited with the NCBI Sequence Read Archive (SRA) under accession number PRJNA648013.

### Quality Control and Transcriptome Assembly

Firstly, Raw reads were processed through in-house Perl scripts developed by the Novogene Bioinformatics Institute (Beijing, China). The clean data were obtained by removing reads that contain adapter or ploy-N and low-quality reads from raw data. Meanwhile, The Phred score (Q20, Q30) and GC content of the clean data were calculated. Reference genome and gene model annotation files were downloaded from National Center for Biotechnology Information (ARS1, https://www.ncbi.nlm.nih.gov/genome/?term=goat). Index of the reference genome was built by Bowtie v2.4.2 and paired-end clean reads were aligned to the reference genome using TopHat v2.1.1. The mapped reads of each sample were assembled by both Scripture (beta2) and Cufflinks (v2.2.1).

### Coding Potential and Conservative Analysis

We used a Coding Potential Calculator (CPC, 0.9-r2) (Kong et al., 2007), Coding-Non-Coding-Index (CNCI, v2) (Sun et al., 2013) and PfamScan (v1.3) (Punta et al., 2012) to assess the protein-coding potential of each novel transcript. CPC examined sequences in NCBI eukaryotes’ protein database and set the e-value “1e-10.” Default parameters were used in CNCI profiles. Pfam searches use default parameters of -E 0.001 -domE 0.001. Candidate sets of lncRNAs that possessed no coding potential were chosen and filtered out from the predicted transcript results from the three tools listed.

### Analysis of Differential Expression

Cuffdiff (v2.2.1) was used to estimate the fragment per kilobase of exon per million fragments mapped (FPKMs) of the transcripts in each sample (Trapnell et al., 2012). The unit of measurement is FPKM. Cuffdiff calculated for the level of expression in each transcript. Gene and transcript expressions based on FPKM values were calculated by Cufflinks transcript quantification engine. Transcripts with a *p* < 0.05 were assigned as differentially expressed.

### Target Gene and miRNA Binding Site Prediction of lncRNAs

To explore the function of candidate lncRNAs, the target genes of candidate lncRNAs were predicted in the cis way. Coding genes 100 k upstream and downstream of lncRNAs were examined as the target genes in cis (co-location genes). The co-expression of coding genes with lncRNAs in different chromosomes were determined with the pearson correlation coefficient (PCC); with PCC >0.95 or < −0.95, the lncRNA-mRNA pair was considered to represent the target genes in trans (co-expressed genes) (Schober et al., 2018). MiRNA binding sites were predicted for the goats with miRanda software, whose principles are based on the miRanda prediction algorithm (Enright et al., 2003). The miRNAs and target lncRNAs were considered when the miRanda score was 140 or higher, and the energy threshold was set to -1.

### Analysis of lncRNA-miRNA Network Interactions

The lncRNA-miRNA interaction network was built according to the prediction of miRNA binding sites. The lncRNA-miRNA interaction analysis was conducted with Cytoscape software (Version 3.6.1). In the network diagram, the connections indicate possible regulatory relationships. The square represents lncRNAs, the circle represents miRNAs, the red represents up-regulated expression and the green represents down-regulated expression.

### Quantitative real‐time PCR Analysis

Total cDNA was synthesized using reverse transcriptase Kit (TaKaRa, Dalian, China). QRT-PCR were performed using CFX96 Touch™ Real-Time PCR Detection System and SYBR^®^ Green PCR Supermixe (Bio-Rad, CA, United States). Each PCR reaction (in 20 μL) involved 10 μL SYBR^®^ Green PCR Supermixe (Bio-Rad, CA, United States), 0.25 μL of each primer, 1 μL cDNA and 8.5 μL H_2_O. The cycling conditions included an initial single cycle (94°C for 3 min), and followed by 40 cycles (94°C for 30 s; 60°C for 30 s; 72°C for 20 s). The primers were designed using Primer 5 (shown in Table 1). The expression level was defined based on the threshold cycle (Ct), and relative expression levels were calculated via the 2^−ΔΔCt^ method. The correlation between the RNA-seq and qRT-PCR results was calculated using the SPSS (Version 18.0.0). *β*-actin was served as internal standard control, and all reactions were performed in triplicate.

**TABLE 1:** The nucleotide sequence used in this study.

ID	Forward sequence (5′–3′)	Reverse sequence (5′–3′)	Length (bp)
XR_001297559.1	TTG​TAC​TCC​GTG​GCC​CTA​AT	CCA​GGC​TAA​TCC​TCC​AAC​C	150
XR_311113.2	TTG​AGA​AAA​CAG​CCA​GTG​C	TAC​CGC​CAG​TGA​CAA​GGA​T	125
XR_001297560.1	TCT​CAT​GCT​AAC​CAG​GAC​CC	AAA​GCC​ACT​GTA​ACC​GCA​CC	150
LNC_000026	GCT​GGA​GTC​TTA​ACT​ATT​GGA​T	ATC​AGA​AAG​GAT​GGG​TGT​G	148
XR_310768.2	AGG​CTT​CCT​CCT​GCT​TGT​G	ATC​CGC​ATC​ATT​TGT​CCA​TT	279
LNC_000155	AGC​CAC​AGT​GAG​CAG​CAT​C	AAA​GGG​AGT​CAT​AGA​GTG​GG	451
XR_001295597.1	ATG​TTC​TTC​ATC​GGC​TTC​ACC	CTC​GTT​CTT​GTC​GTA​GTC​CCA​C	177
β-actin	GTC​ACC​AAC​TGG​GAC​GAC​A	AGGCGTACAGGGACAGCA	208
chi-miR-424-5p mimics	CAG​CAG​CAA​UUC​AUG​UUU​UGA		
chi-miR-3955-5p mimics	UUU​GAU​GGC​UGA​UCC​UCU​CAC​U		
NC	UUC​UCC​GAA​CGU​GUC​ACG​UTT	ACG​UGA​CAC​GUU​CGG​AGA​ATT	
pmiRGLO-1	GGG​TTT​AAA​CCT​TGG​AGC​ATA​GTC​TAC​AGC​A	CCG​CTC​GAG​TCA​TAC​TGA​CCG​ACT​TGT​GC	1005
pmiRGLO-2	GGG​TTT​AAA​CAG​AGA​GGC​ATC​CTT​GTC​ACT​G	CCG​CTC​GAG​AAT​TCC​AAC​AGG​CAA​TCG​TT	1525
chi-miR-135a mut	ACC​CTT​TGA​GGG​GGA​TTG​CTC​GGC​TGA​ATC​C	GGA​TTC​AGC​CGA​GCA​ATC​CCC​CTC​AAA​GGG​T	1005
chi-miR-424-5p mut1	TGC​CAT​ATT​GGG​CAT​CAT​CAT​AGG​GCC​AAA​G	CTT​TGG​CCC​TAT​GAT​GAT​GCC​CAA​TAT​GGC​A	1005
chi-miR-424-5p mut2	AGG​AGA​GAA​GAA​CAT​CAT​CTT​GTC​ATT​GTA​G	CTA​CAA​TGA​CAA​GAT​GAT​GTT​CTT​CTC​TCC​T	1525
chi-miR-544-5p mut	CTA​CCG​GCT​GGA​CGG​TGG​ACC​ACC​ATC​TCA​G	CTG​AGA​TGG​TGG​TCC​ACC​GTC​CAG​CCG​GTA​G	1005
chi-miR-3955-5p mut	CCC​AGC​AAG​AGA​TTG​CTG​GTC​ACC​CTC​TGG​G	CCC​AGA​GGG​TGA​CCA​GCA​ATC​TCT​TGC​TGG​G	1525

Note: The part highlighted with gray was enzyme site induced.

### Plasmid Construction

Two fragments of XR_311113.2 sequence were amplified using I-5™ 2 × High-Fidelity Master Mix (Tsingke, Wuhan, China). All the PCR products were inserted into pmirGLO vector (Promega, WI, United States), respectively. The primers are listed in Table 1. Then two recombinant plasmids were digested with *Pme*I and *Nhe*I (Thermo Scientific, WLM, United States). All constructs were sequenced by Sangon Biotech Co., Ltd. (Shanghai, China). The mutants of binding sites were generated using a MutanBEST Kit (TaKaRa, Dalian, China) and mutagenic primers (shown in Table 1).

### Cell Culture, Cell Transfection, and Dual Luciferase Reporter Assays

The goat kidney epithelial cells (GK cells) were obtained from the Kunming cell bank of the Chinese academy of sciences. Cells were seeded at a density of 1.5 × 10^5^/ml using Dulbecco minimum essential medium (DMEM) (Hyclone, UT, United States) supplied with 10% fetal bovine serum (FBS) medium (Hyclone, UT, United States). The miRNA mimics and negative control (NC) were synthesized by Ribobio (Ribobio, Guangzhou, China). The sequences used are shown in Table 1. Cells were seeded in 24-well plates at a density of 1.5 × 10^5^ cells per well 24 h before transfection. The cells were co-transfected with a mixture of 250 ng pmirGLO recombinant vector plasmid and 3 μL miRNA mimics or NC per wells using lipofectamine 3000 (Life Technologies, MD, United States). The luciferase activity in the transfected cells was then measured after 24 h according to the manufacturer’s instructions with a VICTOR™ X3 PerkinElmer 2030 Multilabel Plate Reader (PerkinElmer, MA, United States).

### Statistical Analyses

Descriptive results of the study are expressed as means ± SD. All data analyses were performed by GraphPad Prism 5 (San Diego, CA). The means of the groups were compared by Student’s t‐test. *p* < 0.05 was considered to indicate statistically.

## Results

### Identification of lncRNAs in Goat Pre-ovulatory Follicles

**FIGURE 1:**
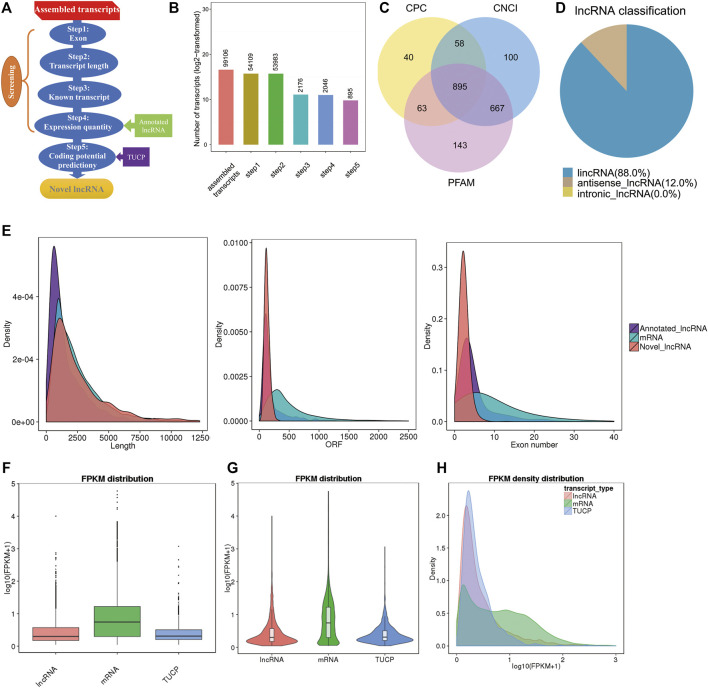
Characterization of pre-ovulatory follicles for goat lncRNAs, mRNAs, and TUCPs. **(A,B)**. Steps of the novel lncRNA screening. **(C)** Coding potency filter with three mainstream coding potential analytical methods (CPC, CNCI, and FFAM). **(D)** Classification of the lncRNAs identified here. **(E)** Transcript size distribution, ORF length, and exon number of lncRNA and mRNA transcripts. **(F−H)** Box plot, violin plot, and density distribution diagram showing the expression features of lncRNAs, mRNAs, and TUCPs in the pre-ovulatory follicles of goats.

To identify the expression of lncRNAs in goat pre-ovulatory follicles, six goats average from two goat breeds, Boer and Macheng goats, were sequenced using an Illumina HiSeq 4000 platform. A total of 41.3 Gb of lncRNA clean data were obtained and mapped to the goat reference genome using TopHat2. We carried out a series of rigorous screening analyses using three analytical software programs (CPC, PFAM, and CNCI) (Figures 1A,B), and a total of 895 lncRNAs from 99,106 assembled transcripts were identified (Figure 1C and Supplementary Table S1). These lncRNAs consisted of 88.0% long intergenic noncoding RNAs (lincRNAs) and 12.0% antisense lncRNAs, with virtually no intronic lncRNAs being detected (Figure 1D). We analyzed the characteristics and expression levels of the lncRNA and mRNA transcripts. As shown in Figure 1E, we found that the predicted lncRNAs were shorter in length than the mRNAs and had fewer exons than the average mRNA. Furthermore, most of the lncRNAs tended to exhibit a relatively shorter open reading frame (ORF) length than the mRNAs. The mRNA levels were significantly higher than those of lncRNAs or transcripts of uncertain coding potential (TUCP) in the pre-ovulatory follicles of goat samples (Figures 1F–H).

### Expression Profiles of lncRNAs and mRNAs in Goat Follicles

**FIGURE 2:**
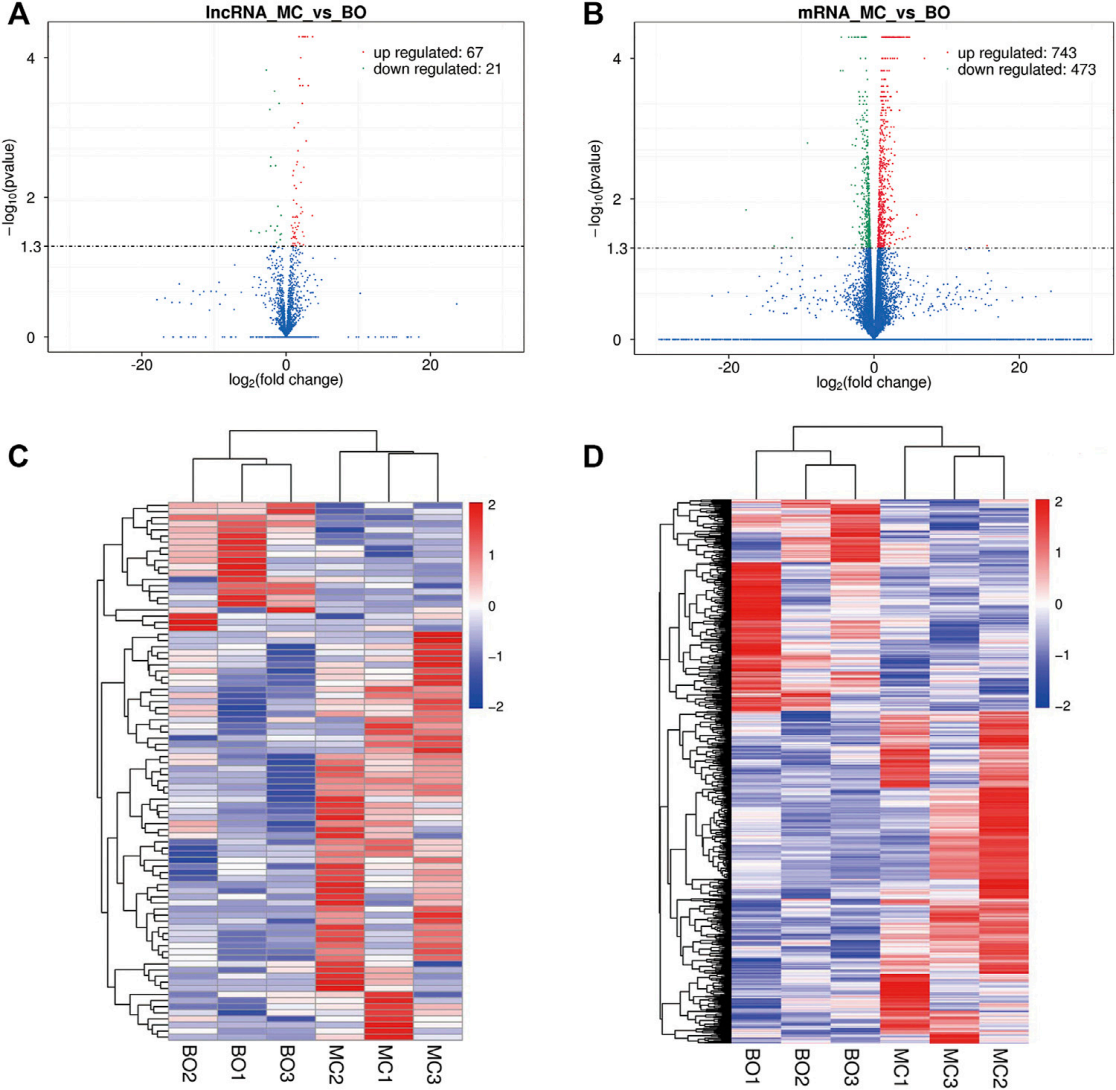
Differential expression of lncRNAs and mRNAs in the pre-ovulatory follicles of goats. **(A,B)** Volcano plots of differentially expressed lncRNA and mRNA transcripts. **(C,D)** Hierarchical clustering of the expression profiles of differentially expressed lncRNAs and mRNAs.

Here, we identified the differential expression profiles of lncRNAs and mRNAs in the pre-ovulatory follicles of Boer and Macheng Black goats. Specifically, a total of 88 lncRNAs (67 upregulated and 21 downregulated) and 1216 mRNAs (743 upregulated and 473 downregulated) were differentially expressed (Figures 2A,B and Supplementary Tables S2, S3). A hierarchical clustering analysis was performed on the differentially expressed lncRNAs and mRNAs, respectively. To gain insight into the similarities of the pre-ovulatory follicles between the Boer and Macheng goats, data from all of the differentially expressed lncRNAs and mRNAs were used in a systematic cluster analysis. The heat map clearly indicated self-segregated clusters in the Boer (BO) and Macheng (MC) groups (Figures 2C,D).

### Overview of Differential lncRNA Expression Profiles

**FIGURE 3:**
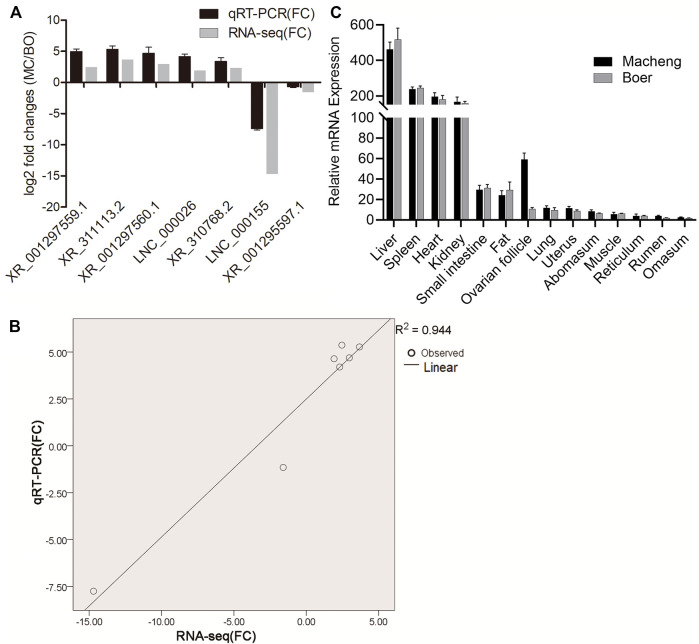
Validation of selected lncRNAs and mRNAs using qRT-PCR. **(A)** Fold changes in the relative expression of lncRNAs, as assessed by qRT-PCR. Results of the comparison of the seven lncRNAs using qRT-PCR and RNA‐seq. The vertical axis indicates the mean fold change (log2-fold change) of each lncRNA. **(B)** Correlation analysis of the fold changes between qRT-PCR and RNA-seq. **(C)** Relative expression of lncRNAs. Expression profile of lncRNA XR_311113.2 in 14 tissues of Macheng and Boer goat expressed as the mean ± SD. Data were normalized to the reference gene (*β*-actin).

Five upregulated (XR_001297559.1, XR_311113.2, XR_001297560.1, LNC_000026, and XR_310768.2) and two downregulated (LNC_000155 and XR_001295597.1) lncRNAs were randomly selected and amplified *via* qRT-PCR to confirm RNA-seq accuracy. As shown in Figure 3A, log2-fold changes (MC/BO) were calculated based on the RNA-seq and qRT-PCR results. The observed expression trends indicated that the two methods produced consistent results. Furthermore, the fold change values measured by RNA-seq and qRT-PCR were significantly correlated (correlation coefficient, 0.944) (Figure 3B). Moreover, we investigated the relative expression levels of the greatly differentially expressed lncRNA XR_311113.2 in 14 goat tissues of Macheng black goat and Boer goat (i.e., liver, spleen, heart, kidneys, small intestine, fat, ovarian follicle, lungs, uterus, abomasum, muscle, reticulum, rumen, and omasum) (Figure 3C). XR_311113.2 tended to exhibit high expression levels in the liver, spleen, heart, and kidneys of Macheng black goat and Boer goat, followed by the small intestine, fat, ovarian follicle, and lungs. By contrast, its low expression was noted in the uterus, abomasum, muscle, reticulum, rumen, and omasum. Notably, the expression level of XR_311113.2 in Macheng ovarian follicle was far greater than Boer, and the fold change was nearly 6. These results demonstrated that there was a general consistency between the qRT-PCR and RNA-seq results, although the fold changes were not exactly the same between the two different technologies. In addition, XR_311113.2 was only significantly dimorphically expressed in ovarian follicle.

### lncRNA-miRNA Interaction Networks

**FIGURE 4:**
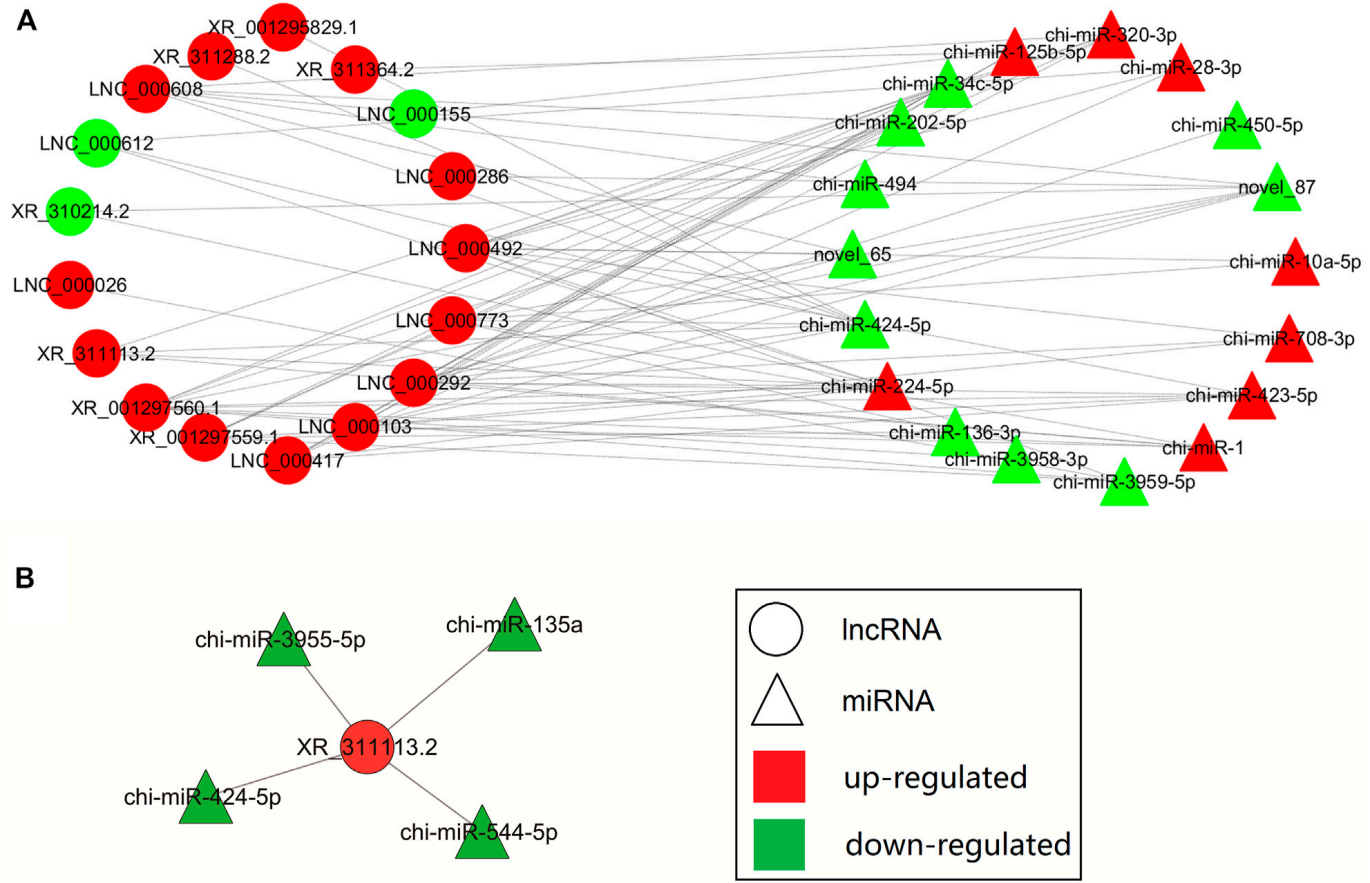
Biomathematically predicted XR_311113.2 targeted in the lncRNA-miRNA network. The networks of lncRNA-miRNA were indicated by Cytoscape. Red: upregulated gene; green: downregulated gene; circle: lncRNA; triangle: miRNA. **(A)** The interaction network of the top 20 differentially expressed lncRNAs and mRNAs. **(B)** Predicted lncRNA-miRNA network of XR_311113.2 with four miRNAs.

The lncRNA-miRNA interaction networks were constructed using the RNA-seq data and potential lncRNA-miRNA connections were explored using the Cytoscape 3.6.1 software (http://www.cytoscape.org/). The lncRNA-miRNA interaction network was established based on the relationship between the differentially expressed (DE) lncRNAs and DE miRNAs. In the network depicted in Figure 4A, we identified the top 20 lncRNAs and top 20 miRNAs as differentially expressed profiles. The top differentially expressed lncRNA XR_311113.2 included binding sites for four miRNAs (chi-miR-135a, chi-miR-424-5p, chi-miR-544-5p, and chi-miR-3955-5p). The interaction network of XR_311113.2 and these four miRNAs was predicted by miRanda (Figure 4B). This result implies that These results indicate that the aforementioned miRNA and lncRNA might have a tight correlation and regulation relationship, which could be our main focus for further study.

The lncRNAs may affect the expression of miRNAs by sponging them.

### XR_311113.2 Serves as a Sponge for Chi-miR-424-5p

**FIGURE 5:**
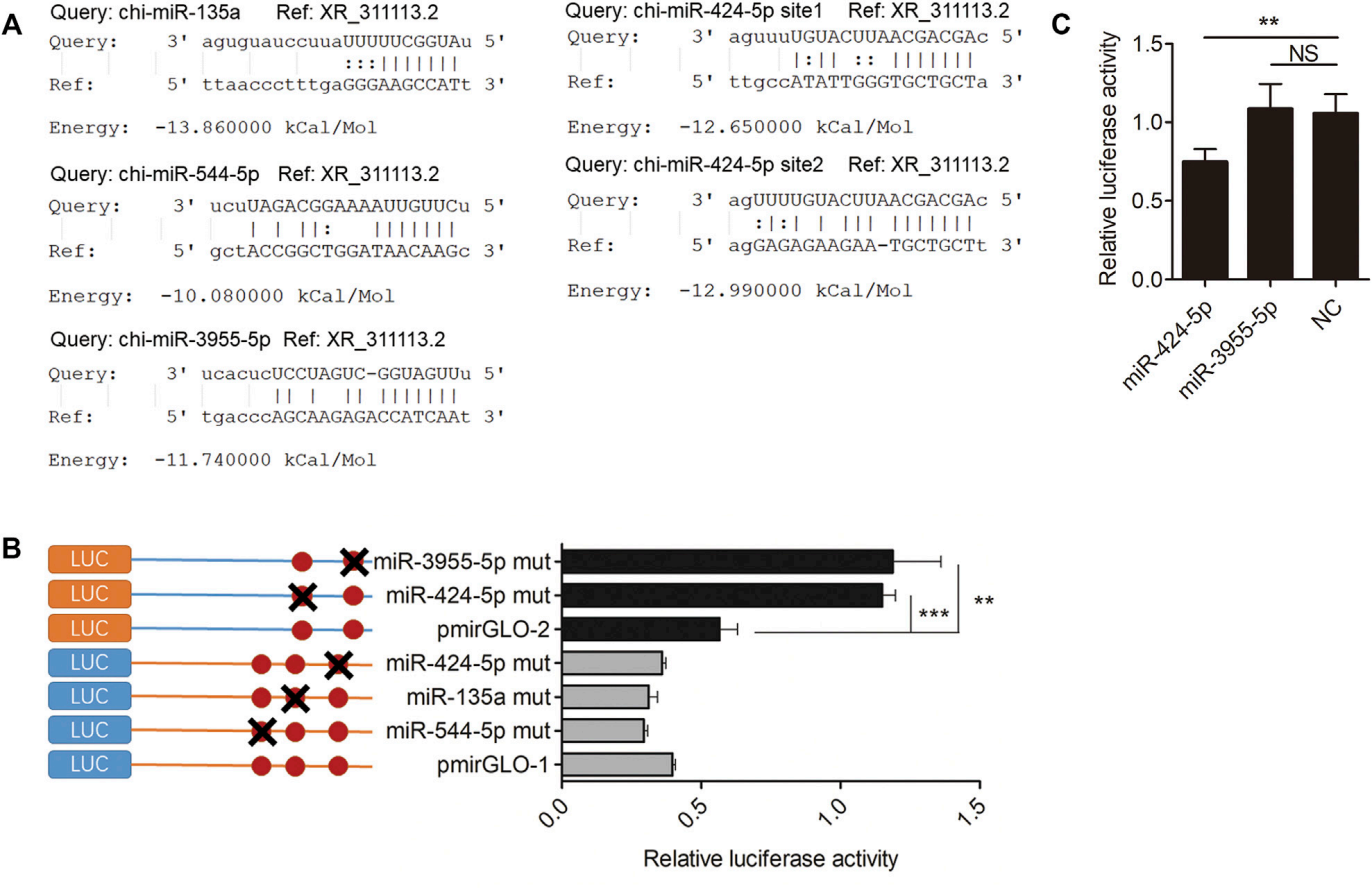
XR_311113.2 serves as a sponge for chi-miR-424-5p. **(A)** Alignment of potential chi-miR-135a, chi-miR-424-5p, chi-miR-544-5p, and chi-miR-3955-5p base pairing with XR_311113.2, as identified by the miRanda software. **(B)** The luciferase activity of miRNA binding sites mutant pmirGLO vector by luciferase assay in GK cells. Site-directed mutagenesis in the chi-miR-135a, chi-miR-424-5p, chi-miR-544-5p, and chi-miR-3955-5p binding site of the XR_311113.2. The pmirGLO-1 and pmirGLO-2 were wild vector of XR_311113.2. **(C)** Luciferase reporter assay to determine the luciferase activity of pmirGLO-2 in GK cells transfected with chi-miR-424-5p and chi-miR-3955-5p mimics, to identify miRNAs that bound to the XR_311113.2 sequence. **p < 0.01; ***p < 0.001; NS, not significant.

The miRanda software was used to predict the putative miRNA binding sites of XR_311113.2. Figure 5A indicates that four miRNAs (chi-miR-135a, chi-miR-424-5p, chi-miR-544-5p, and chi-miR-3955-5p) bind to XR_311113.2 *via* putative binding sites. To verify that these four miRNAs bind to XR_311113.2, the miRNA binding site regions and the mutated regions of chi-miR-135a (719 bp/741 bp), chi-miR-424-5p (975 bp/995 and 3197 bp/3216 bp), chi-miR-544-5p (680 bp/700 bp), and chi-miR-3955-5p (3569 bp/3591 bp) were cloned into two pmirGLO vectors, named pmirGLO-1 (159 bp/1163 bp, 1005 bp) and pmirGLO-2 (2104 bp/3628 bp, 1525 bp), respectively. Three miRNA mutants (chi-miR-544-5p mut, chi-miR-135a mut, and chi-miR-424-5p mut) of pmirGLO-1 and two miRNA mutants (chi-miR-424-5p mut and chi-miR-3955-5p mut) of pmirGLO-2 were transfected into GK cells. We discovered that chi-miR-424-5p mut (*p* < 0.001) and chi-miR-3955-5p mut (*p* < 0.01) completely abolished the suppression of luciferase activity compared with the wild-type vector (Figure 5B). These results suggest that chi-miR-424-5p and chi-miR-3955-5p bind to the putative miRNA binding site of XR_311113.2. To clarify further the binding activity of miRNAs, the mimic of chi-miR-424-5p and chi-miR-3955-5p was cotransfected with the pmirGLO-2 luciferase reporter plasmid into GK cells. Compared with the NC group, chi-miR-424-5p significantly reduced luciferase activity (*p* < 0.01) (Figure 5C). These results suggest that XR_311113.2 serves as a sponge of chi-miR-424-5p.

## Discussion

Goat is a domestic animal with a long history. The Macheng black goat is a unique breed indigenous to mountainous areas of Central China Region and characterized by systemic black and high prolificacy. Boer goat is one of the most popular breeds of meat goat in the world due to their excellent carcass qualities and high growth efficiency (Naing et al., 2010). The reproductive performance of ewes is an important part of goat productivity. The kidding and fecundity rates of Boer goats are approximately 189% and 210% (Malan, 2000). The Macheng black goat is highly fertile, the kidding rate and fecundity rate are approximately 219% and 346% (Tao et al., 2018). Recent studies indicate that a high ovulation rate can increase the probability of higher litter size (Wang et al., 2020). As a high propagation capability goat breed, how lncRNAs regulate its follicular development is still unclear. Therefore, we investigated the differential expression of lncRNAs in ewe follicular development between Macheng goat and Boer goat using RNA-seq.

Folliculogenesis is a complex process that includes primordial follicle recruitment, granulosa cell (GC) proliferation, oocyte maturation, steroidogenesis, and ovulation (McLaughlin and McIver, 2009; Field et al., 2014). GCs play a crucial role in each stage of folliculogenesis, provide nutritional support for the development of oocytes through the gap junction, and enhance the maturation of oocytes. The interaction between GCs and theca cells is an important condition for maintaining follicular function and promoting normal development. The growth and differentiation processes of GCs are key criteria for the normal initiation and growth of primordial follicles, and mediate the development and atresia process of growth-period follicles, thereby playing an important regulatory role in the development of follicles (Uyar et al., 2013; Johnson, 2015; Sugiyama et al., 2016; Nguyen et al., 2019; Kim et al., 2020).

LncRNAs are newly discovered ncRNAs that have important regulatory functions in a range of biological events (Peng et al., 2017), especially their role as therapeutic targets and diagnostic biomarkers of various diseases (Ji et al., 2003; Yan et al., 2015; Huang et al., 2017; Huang, 2018; Simion et al., 2019; Yan et al., 2019). A growing body of evidence has shown that lncRNAs are widely distributed in mammals and plants (Necsulea et al., 2014; Wang et al., 2014; Zhao et al., 2018; Sarropoulos et al., 2019), but little has been reported regarding the folliculogenesis process of goats. In the field of molecular biology, the genetic research of goats is subject to many restrictions compared with the research of other species, such as humans and mice. The database of lncRNAs mainly targets common research subjects (humans, mice, etc). The lack of various databases can hamper the analysis of lncRNA data.

In the current study, we used high-throughput sequencing to analyze the lncRNA profiles in goat ovarian follicle samples. In addition, we investigated the manner in which lncRNA and mRNA networks contribute to follicle development, and confirmed the regulatory function of lncRNA XR_311113.2. We predicted a total of 895 lncRNAs in samples of pre-ovulatory follicles from Macheng black goats and Boer goats, which was less than that detected in the developmental skeletal muscle of fetal goat (3981) (Zhan et al., 2016) and the fetal skin of goats (1336) (Ren et al., 2016), and more than that detected in the anagen-phase skin samples of cashmere goats (437) (Zheng et al., 2019). These results revealed that the expression of lncRNAs was tissue-specific in different goat breeds.

A recent study has revealed that lncRNAs can function as miRNA sponges to further affect the expression of miRNA target genes (Du et al., 2016). For example, lncRNA TRPM2-AS acts as the sponge of miR-612 to promote gastric cancer progression (Xiao et al., 2020), and lncRNA MDNCR binds to miR-133a, thus promoting cell differentiation in bovine primary myoblasts (Li et al., 2018). In the present study, we found that lncRNA XR_311113.2 was differentially expressed in the pre-ovulatory follicles of the Boer and Macheng goat groups. The bioinformatics analyses showed that four miRNAs potentially interacted with XR_311113.2 (as shown in Figure 4). In addition, we validated the interacting relationship between XR_311113.2 and chi-miR-424-5p using luciferase activity assays (as shown in Figure 5). Therefore, our results suggest that XR_311113.2 targets chi-miR-424-5p directly by functioning as a sponge in the pre-ovulatory follicles of goats. Moreover, the previous study results demonstrated that miR-424-5p plays an important role in diseases of the reproductive system by directly targeting several key functional genes (Xu et al., 2013; Liu et al., 2018). In addition, miR-424 suppresses the proliferation and promotes the apoptosis of human ovarian granulosa cells (Du et al., 2020). These observations suggest that lncRNAs regulate goat ovarian follicular development by binding to miRNAs that regulate target gene expression.

One result we find out is really interesting. In Figure 5B, when we mutated the binding site of miR-3955-5p in XR_311113.2 sequence, the luciferase activity of the mutant pmirGLO vector significantly increased compared with wild type pmirGLO vector. The results showed that the mutant site might prevent miR-3955-5p bind to pmirGLO, so that the luciferase activity of pmirGLO will not be inhibited by miR-3955-5p. To clarify further the binding activity of miR-3955-5p, the chi-miR-3955-5p mimic was synthesized and co-transfected with the wild pmirGLO plasmid. Compared with the NC group, chi-miR-3955-5p did not significantly reduce luciferase activity of wild pmirGLO. Why? The reason may be that the mutated binding site is not necessarily the factual site of miR-3955-5p, but may be the binding site of other unknown miRNAs. When this fake site is mutated, the unknown miRNA cannot inhibit the luciferase activity. This is why we continue to use the results of Figure 5C to further eliminate the fake results of Figure 5B and ensure the reliability of the results.

MiRNAs have emerged as key post-transcriptional regulators of target expression through complementary base pairing with the target (Filipowicz et al., 2008). A large number of computational prediction tools for the prediction of putative miRNA targets have been developed, and commonly used tools in mammals include miRWalk, PicTar, miRanda, Targetscan, RNAhybrid, PITA, miRmap, DIANA-microT and RNA22V2 (Riffo-Campos et al., 2016). However, the described tools are mainly developed for a few species such as humans, mice, pigs etc. Some of these tools can only set a few parameters and cannot enter sequences. For obscure livestock such as goats, it is impossible to accurately predict the targeting relationship between miRNA and target. The lack of various databases and tools can make it difficult to carry out the study of goats.

## Conclusion

In conclusion, we analyzed the lncRNA and mRNA expression profiles of goat ewe pre-ovulatory follicles to predict the interactions between lncRNAs and miRNAs. Overall, 895 lncRNAs were identified, 88 of which were preliminarily determined to show a marked differential expression, indicating potentially substantial effects on goat ovarian follicles. The tissue expression profile of lncRNA showed tissue specificity. LncRNA XR_311113.2 may function as a sponge of chi-miR-424-5p. Our RNA-seq data contributed to the known types of lncRNA species. LncRNAs may represent a promising new field of research in the area of ovarian follicular development.

## Data Availability

Sequencing data were deposited in the NCBI Sequence Read Archive (SRA) under accession number PRJNA648013.
